# Loading-Controlled Photoactivity in TiO_2_@BiVO_4_ Heterostructures

**DOI:** 10.3390/molecules31020353

**Published:** 2026-01-19

**Authors:** Małgorzata Knapik, Wojciech Zając, Agnieszka Wojteczko, Anita Trenczek-Zając

**Affiliations:** 1 Faculty of Materials Science and Ceramics, AGH University of Krakow, Al. Mickiewicza 30, 30-059 Krakow, Polandagdudek@agh.edu.pl (A.W.); 2 Faculty of Energy and Fuels, AGH University of Krakow, Al. Mickiewicza 30, 30-059 Krakow, Poland; wojciech.zajac@agh.edu.pl

**Keywords:** TiO_2_@BiVO_4_ heterostructures, photoanodes, water oxidation, SILAR, drop-casting

## Abstract

In this study, we have investigated heterostructural TiO_2_/BiVO_4_ anodes to determine the effect of the amount and form of BiVO_4_ nanoparticles on TiO_2_ on the response of photoanodes under UV and visible illumination. BiVO_4_ nanopowders were prepared and annealed at temperatures ranging from 200 to 500 °C. Structural and optical characterization indicates that as the annealing temperature is increased, a phase transition from a weakly ordered to a dominant monoclinic BiVO_4_ phase is observed, which is accompanied by an increase in visible light absorption. Subsequently, the most crystalline powder was utilized to deposit BiVO_4_ on nanostructured TiO_2_ either as a compact overlayer (drop-casting) or as a progressively grown nanoparticle (TiO_2_@S series) in the successive ionic layer adsorption and reaction process (SILAR). Photoelectrochemical measurements were performed, revealing a morphology-dependent photocurrent response under UV and visible illumination. A further increase in the number of cycles systematically increases the photocurrent in the visible light range while limiting the response to UV radiation. The TiO_2_@d photoanode demonstrates the highest relative activity within the visible range; however, it also generates the lowest absolute photocurrent, indicating the presence of significant transport and recombination losses within the thick BiVO_4_ layer. The results demonstrate that the presence of BiVO_4_ nanoparticles on TiO_2_ exerts a substantial influence on the separation of charge between semiconductors and the synergistic utilization of photons from the UV and visible ranges. This research yielded a proposed scheme of mutual band arrangement and charge carrier transfer mechanism in TiO_2_@BiVO_4_ heterostructures.

## 1. Introduction

Solar-based water splitting is one of the promising routes for sustainable hydrogen production and chemical energy storage. Among the various approaches to convert sunlight into chemical fuels, photoelectrochemical (PEC) water splitting demonstrates a simple and practical approach. In PEC gas evolution reactions in an aqueous electrolyte under bias-assisted light are directly induced by semiconductor photoelectrodes [1,2,3]. Metal-oxide photoanodes have a particular feature, and are promising materials due to the Earth-abundant elements, good chemical stability and compatibility with low-cost processing. But such material is hindered technically; their implementation is not yet easy owing to wide band gaps, limited absorption of visible light, short carrier diffusion lengths, and pronounced interfacial recombination losses [1,4,5].

Titanium dioxide (TiO_2_) is still among the most studied oxide photoanodes. It is characterized by chemical robustness, low toxicity, structural versatility, and its surface chemistry and defect physics have been well described [6,7,8,9]. In particular, thermally, chemically and electrochemically prepared TiO_2_ nanostructured layers on titanium foils reveal a highly developed and diversified surface morphology, resulting in better photoelectrochemical performance [10,11,12]. For these reasons, they are attractive as electron-transport substrates and scaffold materials for visible-light absorbers. The wide band gap (3.0–3.2 eV) of TiO_2_, however, limits its absorption mostly to the ultraviolet (UV) region. Thus, unmodified TiO_2_ generally shows negligible photocurrent under visible-light illumination. This makes its efficiency in water splitting process affected only by UV photons. This drawback has led to various strategies to enhance the spectral response of TiO_2_ or to enhance its charge separation efficiency, such as doping, surface sensitization, and the development of heterostructures with narrower-band-gap semiconductors [7,11,12,13,14,15,16,17,18].

Among the visible-light responsive semiconducting photoanodes, bismuth vanadate (BiVO_4_) has been presented as a promising candidate in solar water oxidation [19,20]. In a broader context, recent advances have rendered photoanodes effective (photo)electrocatalytic interfaces for activating molecular oxygen and generating ROS to reduce pollution. These advances include oxygen activation mechanisms, oxidant-assisted PEC architectures, and visible-light-utilizing photoanode materials designed for reliable operation in wastewater treatment [21]. Numerous polymorphs of BiVO_4_ have been reported for their relative stability and electronic properties [22,23,24,25,26,27]. It is through them that the monoclinic scheelite phase merges a well-matched band gap (2.4–2.5 eV) and useful charge transport properties that underwrite its use in photocatalysis and photo-electrochemical water oxidation [19,28,29]. On the other hand, BiVO_4_ also suffers from intrinsic characteristics, including short hole diffusion length, modest bulk conductivity, and a strong sensitivity of its optoelectronic properties to synthesis conditions, phase composition, and defect chemistry [30,31,32,33]. The same nominal BiVO_4_ structure can be characterized with very different degrees of structural order, light scattering, and band-edges as a result of the course of preparation and thermal history. When mixing BiVO_4_ with a TiO_2_ into heterostructure, the PEC response indicates not only the new visible-light absorption but also the separation of charge carriers between components of heterostructure.

The TiO_2_/BiVO_4_ pair is therefore a well-studied heterostructural photoanode. Numerous studies indicate an increase in the value of generated photocurrent and better efficiency of solar energy conversion into hydrogen. This can be attributed to a favorable interfacial energetics reported for TiO_2_/BiVO_4_ and effective charge separation at the interface [20,34,35,36,37]. At the same time, there is no consensus on the exact band configuration and charge transfer mechanism: both the classic type II configuration and the Z- and S-schemes have been proposed. The reported positions of the band edges of BiVO_4_ are sensitive to surface states and crystal structure defects, which explains why band diagrams may differ between studies. For instance, density functional theory (DFT) analysis for BiVO_4_(001) places the E_VB_/E_CB_ near −6.8/−4.6 eV (relative to the vacuum) [38]. Recent engineering work on the rutile TiO_2_/monoclinic BiVO_4_ interface further demonstrates that effective alignment and charge transfer pathways may depend on the junction structure (e.g., BiVO_4_ facet/orientation). Mott-Schottky and EIS have been used to rationalize when the type II pathway becomes favorable [39]. Analogous arguments concerning band shift and built-in field have been invoked for other BiVO_4_-based heterostructures (e.g., g-C_3_N_4_/BiVO_4_), underscoring the notion that charge separation is governed by the relative arrangement of VB and CB levels at the phase boundary, rather than solely by the nominal bandgap energies [40]. This debate is further complicated by the fact that actual TiO_2_/BiVO_4_ photoanodes are not ideal. These “imperfections” have a significant impact on light scattering, effective optical path length, and local electric fields. All of this means that they have a significant impact on energy conversion efficiency.

However, the combination of BiVO_4_ with TiO_2_ has been observed to result in certain complications. The presence of thick, continuous layers of BiVO_4_ has been shown to have a shielding effect on TiO_2_, preventing it from being exposed to the electrolyte and light. This phenomenon leads to the transportation of photogenerated charge carriers over considerable distances. However, in the case of thin and discontinuous layers or nanometer-sized separated BiVO_4_ particles, the situation is different. Local TiO_2_-BiVO_4_ connections, with access to light and electrolyte through TiO_2_, enable the interaction of both semiconductors. This prompts the fundamental question of how the morphology of deposited BiVO_4_ affects the ratio of activity in UV radiation to visible light.

In this study, we have prepared TiO_2_@BiVO_4_ heterostructure electrodes as a model system for the systematic investigation of the influence of structural ordering, morphology, and deposition conditions of BiVO_4_ on TiO_2_ on the properties of the photoanode. In light of the preceding research on TiO_2_-based heterostructures [41,42,43,44,45,46], thermally deposited TiO_2_ layers on etched Ti foils [10] and BiVO_4_ homostructures, the present study investigates the impact of the deposition of BiVO_4_ on a TiO_2_ substrate with complex morphology on the equilibrium between light absorption, charge transport, and interfacial recombination in TiO_2_@BiVO_4_ photoanodes. The process of growing nanostructured TiO_2_ layers directly on a Ti foil is accomplished through the utilization of chemical etching and thermal oxidation in a straightforward two-step procedure. This ensures mechanical stability, complex crater-like morphology, electron transport, and a UV-absorbing matrix. In this substrate, BiVO_4_ particles of varying crystallinity are deposited by drop-casting and successive adsorption and reaction of ionic layers (SILAR). The photoanodes obtained by this method were then subjected to structural analysis, with the band gap energy being determined. Following this, tests were carried out in a photoelectrochemical cell. This research yielded a probable mechanism for charge carrier transfer between the components of the heterostructures.

## 2. Results

### 2.1. Bismuth Vanadate Powders

**Figure 1:**
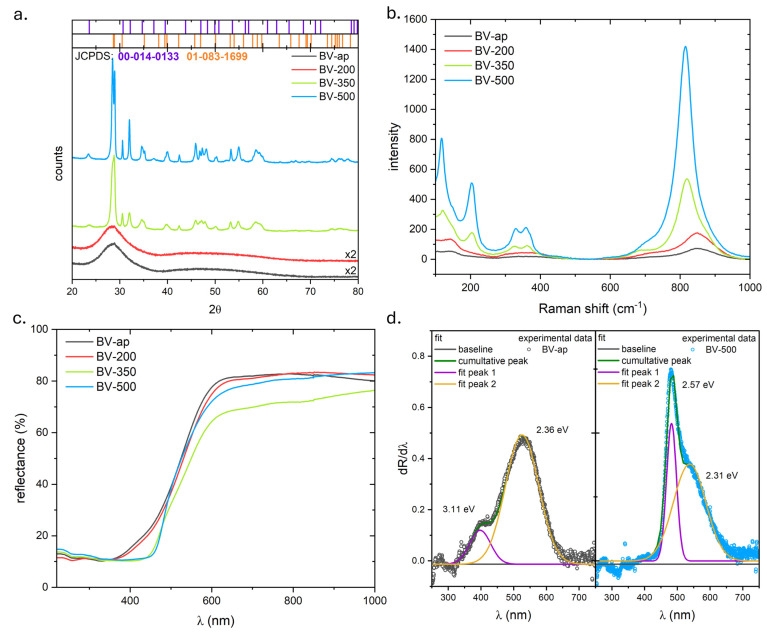
XRD (**a**), Raman (**b**) and reflectance (**c**) spectra as well as differential analysis of band-gap energy (**d**) of as-prepared and annealed BiVO_4_ powders. The top of 1a: JCPDS 01-083-1699 and 00-014-0133 patterns.

As illustrated in Figure 1a, the outcomes of X-ray diffraction analysis and Raman spectroscopy for BiVO_4_ powders subjected to different thermal treatments are presented. In the case of the as-prepared sample and the sample that was subjected to annealing at 200 °C, the diffractograms show only a broad halo in the 2θ range of approximately 24–35°. The absence of distinct diffraction peaks indicates an amorphous nature. Annealing at 350 °C has been shown to result in significant changes when compared to the temperature of 200 °C. The presence of diffraction reflections is indicative of the crystallization of the powder. No significant changes were observed in the sample that had been annealed at 500 °C. Phase analysis demonstrated the presence of two polymorphic forms of BiVO_4_: monoclinic and tetragonal. The monoclinic phase was predominant (approximately 87%) at 500 °C. However, as demonstrated by the diffractogram, it is challenging to ascertain whether the tetragonal phase crystallizes in the I41/amd or I41/a system, owing to the convergence of the positions of the reflections of both phases. However, the crystallite size increases with temperature; at 350 °C, the values are 19.2 nm (monoclinic) and 13.6 nm (tetragonal), while at 500 °C, they reach 46.1 nm and 37.3 nm, respectively. The crystal structure parameters are outlined in Table 1.

As illustrated in Figure 1b, the Raman spectra analysis demonstrates that an increase in the annealing temperature of BiVO_4_ results in an enhancement in signal intensity, accompanied by band shifts and the emergence of new bands. For the BV-ap and BV-200 powders, maxima are observed at 106.2 and 140.9 cm^−1^, along with broad bands between 280–486 cm^−1^ and 600–1000 cm^−1^. It has been demonstrated that powders which have been subjected to annealing at elevated temperatures exhibit well-defined, separate maxima in these areas. This finding is in accordance with the XRD results, suggesting the presence of an amorphous phase. Conversely, the results of X-ray analysis suggest the presence of monoclinic and tetragonal phases, whose contribution to the amorphous phase must be below the detection threshold of the XRD method.

For samples BV-350 and BV-500, bands are observed at approximately 120.3, 150.1, 203.7, 324.8, 361.7, 710.0, 775.0, 815.0, and 847.0 cm^−1^. The bands observed in the range of 300 cm^−1^ and below can be attributed to lattice (external) modes. These modes correspond to translations/rotations of structural units, vibrations of Bi and V cations in their lattice sites, and collective lattice motions [22,23]. The bands between 300 and 400 cm^−1^ correspond to the ν2 bending of VO_4_ units. The presence of bands in the range of approximately 325 cm^−1^ and 367 cm^−1^ indicates the presence of some distortions in the VO_4_ tetrahedron [23].

The band at approximately 710 cm^−1^ is attributed to the antisymmetric V-O stretching mode of the monoclinic phase. The maximum near ~815 cm^−1^ is attributed to ν1 (symmetric V-O stretching) of the monoclinic phase, although it is shifted downwards from the typical position of ~826 cm^−1^ (probably due to deformation/nanocrystallinity). The bands observed at approximately 770 cm^−1^ and 850 cm^−1^ are attributed to the antisymmetric (ν3) and symmetric (ν1) stretching of V-O bonds in the tetragonal phase, respectively [23]. The bands at 710 and 826 cm^−1^ coexisting with the tetragonal bands at 759 and 855 cm^−1^ serve to confirm the existence of mixed phases [23]. In addition to the primary monoclinic V-O mode of BiVO_4_ at around 827 cm^−1^, a weak high-wavenumber arm is observed in the range of 880–890 cm^−1^. It has been demonstrated that analogous features have been observed in association with the orthorhombic BiVO_4_ polymorph [22,23]. However, this phase is not expected to be stable under the synthesis conditions employed in this study [24,25,26] and is not detected by XRD. The distorted local structure of the monoclinic scheelite phase has been comprehensively documented in structural studies [27]. Consequently, this band can be attributed to locally distorted VO_4_ units and/or short-range structural disturbances within the monoclinic/tetragonal matrix, rather than to the rhombic phase. Confocal microscopy images were also captured during the measurements. The images and their analysis are presented in Figure S2 in the Supplementary Materials.

As illustrated in Figure 1c, the spectral dependence of the total reflectance of bismuth vanadates is evident. As is evident, the shapes of the BV-ap and BV-200 curves are essentially the same. In the case of the BV-350 sample, a change in the slope of the curve can be observed in the range of 450–500 nm, which is even more pronounced in the case of the BV-500 powder. The energy band gaps of the phases forming BiVO_4_ powders were determined using the differential method (Figure 1d) based on the reflection spectra R(λ). For each sample, the presence of two band gap energies was confirmed, thereby demonstrating the existence of two crystalline phases. This finding is consistent with the outcomes of the Raman spectrum analysis, which indicated the presence of monoclinic and tetragonal phases in the BV-ap and BV-200 powders. The E_g_ values are 2.36 and 3.11 eV, and 2.35 and 3.07 eV, respectively. For powders that have undergone annealing at elevated temperatures, the band gap energy for BV-350 powder is measured at 2.30 eV for the monoclinic phase and 2.63 eV for the tetragonal phase. For BV-500, these values are 2.31 and 2.57 eV, respectively. The band gap energies of BiVO_4_ powders are shown in Table 1.

A thorough analysis of the E_g_ values indicates that both BV-ap and BV-200 exhibit a larger energy gap than the tetragonal crystalline form (2.9 eV). This phenomenon can be attributed to the constrained arrangement of the BiVO_4_ crystal lattice, as evidenced by the presence of the Urbach tail in the spectrum (Figure 1c). The Urbach tail is characterized by a blurring of the fundamental absorption edge at shorter wavelengths. Following the annealing process at temperatures of 350 and 500 °C, a notable enhancement in the sharpness of the edge is observed, attributable to the elevated crystallinity of the powder [19,28]. Furthermore, an energy gap of approximately 2.6 eV is evident for both the BV-350 and BV-500 powders. This energy is considerably lower than expected for the tetragonal phase I41/amd and is indicative of the tetragonal phase I41/a. This finding suggests that an annealing temperature of 350 °C is adequate to induce the phase transition from I41/amd to I41/a. Consequently, the analysis of spectrophotometric measurements facilitated the determination of the phase composition, a feat that was not fully achieved through XRD and Raman spectroscopy.

BV-500 powder exhibits a crystalline structure, phase composition, and morphology that renders it particularly well-suited for photoactive applications. A comparison of this sample with others reveals a higher degree of crystallinity, an increased percentage of the monoclinic phase, and an absorption spectrum that encompasses a broader range of wavelengths. Consequently, it was selected for deposition on TiO_2_ using the drop-casting method.

### 2.2. TiO_2_@BiVO_4_ Heterostructures

**Figure 2:**
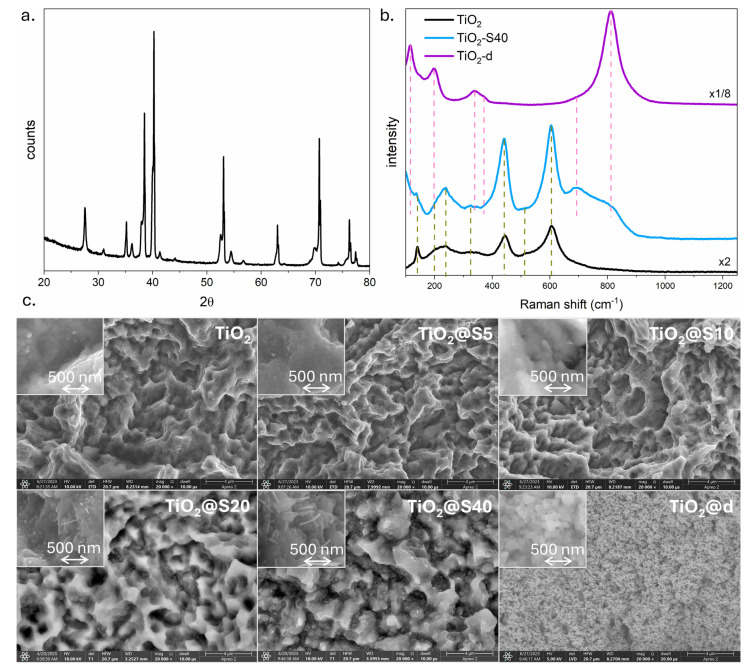
XRD pattern of TiO_2_ (**a**), Raman spectrum (**b**) and SEM images (**c**) of TiO_2_ substrate and TiO_2_@BiVO_4_ heterostructures. Dashed line in (**b**): green—position of TiO_2_ anatase Raman modes, red—position of BiVO_4_ Raman modes.

**Figure 3:**
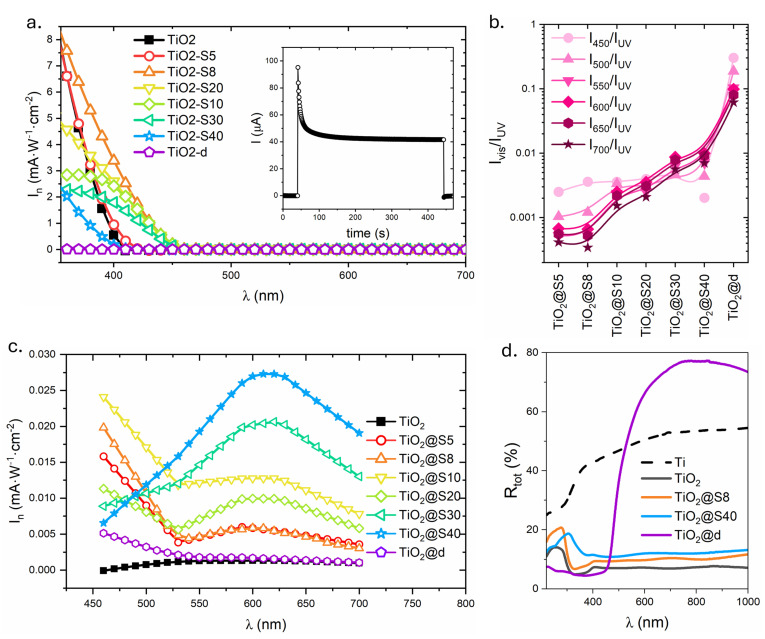
Spectral dependences of TiO_2_ and TiO_2_@BiVO_4_ heterostructures: (**a**) normalized photocurrent characteristics in the UV-vis range with the exemplary current-time curve used to determine I_n_ in the inset; (**b**) ratio of the photocurrent in visible light to the photocurrent in UV (I_vis_/I_UV_) for selected wavelengths; (**c**) normalized photocurrent characteristics in the visible-light range; (**d**) the total reflectance.

A thorough analysis of the X-ray diffractogram of TiO_2_, obtained by subjecting etched titanium foil to oxidation, revealed the presence of diffraction peaks originating from α-Ti, rutile TiO_2_, and lower titanium oxide Ti_6_O (Figure 2a). The predominance of the signal stemming from the substrate suggests that the oxide layer is comparatively thin, given its penetration depth relative to that of X-rays. In the case of TiO_2_@BiVO_4_ samples, no signal originating from BiVO_4_ was detected, which is consistent with the small amount of BiVO_4_, i.e., below the practical detection limit of XRD in thin surface coatings. Consequently, the findings from Raman analysis and UV-vis-NIR spectrophotometry offer substantial evidence for monitoring the contribution of BiVO_4_ in these heterostructures (see Figure 2b and Figure 3d).

As illustrated in Figure 2b, the Raman spectra of the TiO_2_, TiO_2_@S40, and TiO_2_@d samples are presented, with the positions of the bands clearly marked. In the case of TiO_2_, seven bands were identified: those assigned to anatase at 140, 202, and 512 cm^−1^ and to rutile at 140, 239, 444, and 607 cm^−1^. The 140-cm^−1^ band can be attributed to two factors. First, it is due to the symmetric O-Ti-O bending in anatase. Second, it is due to the translational vibrations of the crystal lattice involving Ti in rutile. The band at 202 cm^−1^ is indicative of internal bending or the crystal lattice in anatase. The combination or overtones present in rutile result in a signal at 239 cm^−1^. The observed mode at 324 cm^−1^ can be attributed to second-order combinations in rutile and lower titanium oxides, such as Ti6O. A mode resulting from flat bending in rutile has been identified at 444 cm^−1^. The symmetric stretching of Ti-O produces a signal at 512 cm^−1^ for anatase and 606 cm^−1^ for rutile, as reported in [47,48,49,50,51]. For samples exhibiting a heterogeneous structure of TiO_2_@S40 and TiO_2_@d, bands attributed to BiVO_4_ were also observed at 116, 197, 338, 370, 692, and 811 cm^−1^. It is noteworthy that the intensity of the bands originating from TiO_2_ and BiVO_4_ varies depending on the specific heterostructure. This outcome is attributable to the disparities in the methodologies employed for the deposition of BiVO_4_ on the TiO_2_ surface. The SILAR method enabled the deposition of smaller amounts of bismuth vanadate compared to the drop-casting method. Consequently, the substantial thickness of the BiVO_4_ layer in TiO_2_@d effectively restricts access to the TiO_2_ surface, thereby significantly attenuating the substrate signal and predominating the Raman modes of BiVO_4_. Conversely, in the TiO_2_@S40 sample, the amount of BiVO_4_ is negligible, resulting in a Raman signal that is predominantly influenced by modes derived from TiO_2_. The detailed positions of the identified Raman modes are listed in Table S1.

Figure 2c presents SEM images of the TiO_2_ substrate used to create the heterostructures and selected TiO_2_@BiVO_4_ heterostructures. In the preparation of TiO_2_, a two-step process was employed, involving chemical etching and subsequent oxidation of the titanium foil. This method yields a surface that is characteristic of the process, exhibiting a crater-like morphology [10]. For up to 10 SILAR cycles, the surface remains analogous to that of the TiO_2_ reference sample, reflecting the minimal amount of BiVO_4_ deposited. In contrast, the surface of TiO_2_@S20 exhibits a distinct difference. The presence of elongated, needle-like BiVO_4_ particles is discernible at the base of the craters, a phenomenon that does not manifest with reduced number of deposition cycles. Furthermore, these particles are also significantly larger and more numerous in the TiO_2_@S40 sample. The surface morphology of TiO_2_@d differs significantly from that of the other samples. The employment of the drop-casting method results in the deposition of a BiVO_4_ layer comprising uniform spherical particles with a diameter of less than 40 nm. In general, a gradual transition from a mostly uncovered TiO_2_ surface (low SILAR cycle number) to increasingly denser BiVO_4_ particles (S20–S40) should increase the density of TiO_2_-BiVO_4_ connections while maintaining open access paths for the electrolyte and light, so-called quadrupole points. Conversely, drop-casting yields a compact layer that maximizes BiVO_4_ surface coverage and visible light absorption. However, this method may also hinder charge carrier transport and increase recombination due to increased effective thickness and reduced permeability. This compromise, resulting from the morphology, directly explains the dependencies observed in the measurement results of TiO_2_@BiVO_4_ photoanodes in a PEC cell (Figure 3).

As Figure 3d illustrates, the spectral dependence of the total reflectance of TiO_2_ and selected TiO_2_@BiVO_4_ heterostructures is evident. For the purpose of comparison, a curve recorded for titanium foil that has not undergone etching or oxidation is also included. As demonstrated in Figure 1, the processes of etching and oxidation result in a substantial reduction in reflectance. Conversely, the gradual accumulation of BiVO_4_ particles during the SILAR process, which remains relatively small even after 40 deposition cycles, results in slight interference effects in the visible range and a moderate increase in reflectance. Conversely, the BiVO_4_ layer deposited by the drop-casting method exhibits a complete coverage of the TiO_2_ surface, and the absorption edge observed between 450 and 600 nm can be attributed to BiVO_4_.

The band gap energy was calculated based on the spectral dependence. The results indicate that the band gap energy is 3.18 eV for TiO_2_, corresponding to anatase, and 2.34 and 2.57 eV for BiVO_4_, consistent with the monoclinic and tetragonal phases, respectively. This apparent discrepancy between the XRD and Raman/optical data can be attributed to the significantly higher sensitivity of Raman spectroscopy and optical methods for phases whose quantity is below the XRD detection limit. The calculations also align with the SEM observations, indicating that the amount of BiVO_4_ deposited by the SILAR method is inadequate to induce the fundamental absorption edge in the reflection spectrum. Conversely, in the case of the drop-casting method, the deposited BiVO_4_ layer is sufficiently thick that the absorption edge originating from TiO_2_ is no longer visible on the spectral curve.

The photoelectrochemical response (photocurrent) of TiO_2_@BiVO_4_ heterostructures was evaluated based on the photocurrent generated in a three-electrode photoelectrochemical cell under the illumination of monochromatic UV-vis radiation. Hereafter, the term “photoactivity” refers to the photocurrent density measured under illumination in the PEC configuration. Figure 3a presents the spectral dependence of the normalized photocurrent density, I_n_ (photocurrent divided by the incident light power density), for all samples. The I_n_(λ) values were extracted from the kinetics of current changes recorded at selected wavelengths, as illustrated in Figure 3a. Figure 3c magnifies the visible light region, where the photocurrent is notably diminished.

The spectra in Figure 3a can be conveniently discussed by separating the UV and visible regions. In the case of TiO_2_, a strong response is exhibited below 400 nanometers, consistent with its wide energy gap, and practically no activity is observed at longer wavelengths. Subsequent to the deposition of BiVO_4_ employing the SILAR method, the UV photocurrent initially increases, reaching a maximum for samples obtained with a limited number of deposition cycles. This phenomenon can be attributed to the enhanced efficiency of charge separation within the newly formed TiO_2_/BiVO_4_ junctions. For a sample that has undergone more than eight cycles, there is a systematic decrease in the intensity of the UV range, which is likely due to the increasing thickness of the BiVO_4_ layer. This layer provides partial shielding for the TiO_2_ surface from light and electrolyte. For the TiO_2_@d sample, the normalized UV photocurrent is negligible, indicating that this configuration is essentially inactive under UV excitation.

Figure 3b presents a comparison of photocurrent coefficients recorded at selected wavelengths in the visible range to photocurrent at 350 nm (I_vis_/I_UV_). This parameter is indicative of the relative contribution of the reaction to visible light in relation to the characteristic excitation by UV light in TiO_2_. In the case of samples obtained after a limited number of SILAR cycles, the I_vis_/I_UV_ values are low, which indicates the dominant role of TiO_2_. With an increase in the number of BiVO_4_ deposition cycles, these coefficients systematically increase at all analyzed wavelengths. This finding suggests a gradual enhancement in the contribution of BiVO_4_ to the generation of photocurrent. In the case of the TiO_2_@d sample, the I_vis_/I_UV_ ratios reach maximum values, which can be attributed to the combination of insignificant UV photocurrent and relatively increased activity in the visible range. This phenomenon can be attributed to the presence of a BiVO_4_ layer, which is likely to be substantial in thickness. This layer functions as an effective absorber, exhibiting limited recombination but deficient charge transport. In summary, these observations demonstrate that as the BiVO_4_ content on the TiO_2_ surface increases, the heterostructures gradually take over both absorption and charge transport under visible light irradiation.

These PEC trends are consistent with the structural and optical characteristics of TiO_2_@BiVO_4_ electrodes. In the SILAR series, the TiO_2_@S40 sample demonstrates the highest photocurrent density within the visible range (see Figure 3b,c), suggesting that the comparatively thin BiVO_4_ coating most efficiently captures photons with wavelengths ranging from approximately 450 to 750 nanometers while maintaining adequate contact with the TiO_2_ surface. This phenomenon is further substantiated by the I_vis_/I_UV_ ratios for varying wavelengths (see Figure 3b), which undergo a systematic increase from TiO_2_@S5 to TiO_2_@S40 as the BiVO_4_ content is elevated. This observation signifies an increasing contribution of the BiVO_4_ component to the overall response. For the TiO_2_@d drop-casting electrode, the I_vis_/I_UV_ ratios reach their highest values because the compact BiVO_4_ surface layer almost completely screens the TiO_2_ and strongly suppresses photocurrent in the near-UV range. However, the absolute photocurrent in the visible range is the lowest in the entire series (Figure 3c), which is consistent with severe transport and recombination limitations in the thick BiVO_4_ layer. Consequently, the ideal equilibrium between light absorption and charge transport properties is attained near the composition TiO_2_@S40, which is in accordance with the morphological image of the BiVO_4_ coating on the TiO_2_ nanostructure, which is matched but not completely blocking.

## 3. Discussion

**Figure 4:**
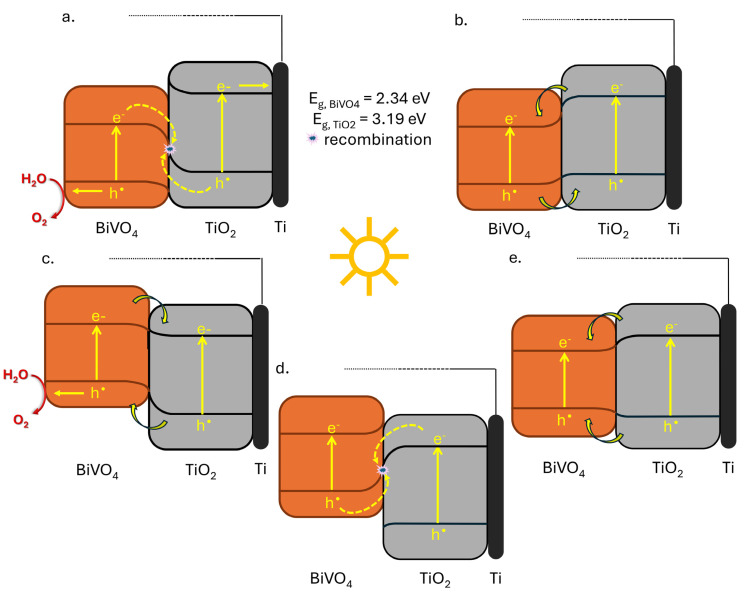
Working schematic band alignment models for the TiO_2_@BiVO_4_ heterostructured photoanode, discussed using literature-reported band positions: S-scheme (**a**,**d**), type II (**b**,**c**) and type I (**e**).

Given the established correlation between PEC performance and interfacial charge separation in TiO_2_@BiVO_4_ electrodes, it is rational to explore the implications of band alignment at the TiO_2_/BiVO_4_ interface. The precise band alignment at this interface remains a subject of ongoing research and discussion. The positions of the bands edges of monoclinic BiVO_4_ exhibit a relatively wide range (E_CB_ < 0.0–0.4 V and E_VB_ ~ 2.4–2.8 V relative to NHE), depending on factors such as the synthesis method, crystal structure, local distortions, or application specifics [19,24,25,29]. In addition, the conduction band edge of anatase TiO_2_ in neutral electrolytes is generally situated between −0.2 and −0.4 V relative to NHE, with a valence band spanning 2.8–3.0 V relative to NHE [10]. Consequently, for TiO_2_/BiVO_4_ heterostructures, a conventional type I, II, as well as Z-scheme and S-scheme has been proposed [20,34,52,53,54]. Distinguishing between the Type II charge-transfer pathway and an S-scheme scenario at the TiO_2_/BiVO_4_ interface requires band-edge positions under electrochemical conditions and insight into interface states. Therefore, the schematic band diagram in Figure 4 is discussed as a working framework, using literature-reported experimental parameters for TiO_2_/BiVO_4_ (e.g., flat-band/work-function and photoelectron spectroscopy data) commonly employed to rationalize Type II alignment in this heterojunction [34,55]. At the same time, Mott-Schottky-derived parameters can be affected by surface capacitance, surface/interface states, and partial Fermi-level pinning, and thus Figure 4 should not be interpreted as an absolute energetic map [55,56,57,58]. In addition to the conventional Type II configuration, step-scheme (S-scheme) charge transfer has been increasingly invoked for BiVO_4_/TiO_2_ heterojunctions to describe selective recombination of low-energy carriers while preserving strongly oxidizing holes and strongly reducing electrons in the respective components [59]. This picture is typically linked to Fermi-level equilibration and a built-in interfacial electric field, and is often supported by evidence related to work function/contact potential differences and by EPR or radical-trapping experiments that connect the proposed charge-flow direction with ROS signatures and activity trends [59,60]. In photoanode studies, S-scheme-type interpretations have also been reported to be consistent with improved PEC metrics in multi-component architectures [61,62,63]. As emphasized in recent methodological reviews, a definitive discrimination between Type II and S-scheme would benefit from complementary probes, in particular EIS (charge-transfer resistance and interfacial capacitance), which has been used to quantify junction-dependent kinetics in related photoanode systems and will be addressed in future work [64,65].

Figure 4a–e summarizes several possible carrier transfer paths considered in this work to assess which scheme is most consistent with our observations. For the sake of clarity, the energy gap values as determined in the present study are herein outlined in conjunction with the representative E_VB_/E_CB_ ranges and redox levels of water as reported in the extant literature. These values can be found in Table S2 (Supplementary Materials) [34,66,67,68]. Figure 4e (type I) is predicated on the assumption that both carriers are transferred from BiVO_4_ to TiO_2_, a supposition that is inconsistent with the insignificant response of TiO_2_ to visible light. Figure 4b and Figure 4d demonstrate the formation of electrons in BiVO_4_ and holes in TiO_2_, respectively. These diagrams are incompatible with the PEC architecture, in which TiO_2_ establishes an electron transport path to Ti and BiVO_4_ interfaces with the electrolyte. Electrons contributing to the external photocurrent must pass through TiO_2_ to the Ti substrate, while holes should oxidize water at the BiVO_4_/electrolyte interface. Consequently Figure 4b,d,e are rejected.

The remaining Figure 4a and Figure 4c, illustrate the placement of electrons in TiO_2_ and holes in BiVO_4_, respectively. This arrangement is consistent with the geometry of the electrode. This observation is also consistent with the increase in photocurrent observed in the visible light range and the I_vis_/I_UV_ ratio for heterostructures. Figure 4c corresponds to a type II connection, involving the transfer of electrons from BiVO_4_ to TiO_2_ and the transfer of holes from TiO_2_ to BiVO_4_. In contrast, Figure 4a represents an S-scheme process, characterized by interfacial recombination of low-energy electrons in BiVO_4_ and low-energy holes in TiO_2_. In light of the prevailing literature on band edges, which typically places the conduction band of TiO_2_ at more negative potentials and the valence band of BiVO_4_ at more positive potentials, the alignment of the S-scheme (Figure 4a) appears to be more consistent with the available data than the ideal type II junction [20,34,52,53]. Consequently, we posit that the S-scheme depicted in Figure 4a most accurately reflects the composition of our TiO_2_@BiVO_4_ photoanodes on a Ti substrate. It is noteworthy, however, that a pure type II mechanism, as illustrated in Figure 4c, cannot be entirely discounted.

Finally, experimental trends are consistent with an interfacial charge-separation scenario discussed using literature-reported band positions (Table S2 in the Supplementary Materials) exhibited in Figure 4a, wherein BiVO_4_ functions as the primary visible light absorber, while TiO_2_ operates predominantly as a UV light absorber and electron transport scaffold. The visible light photocurrent and I_vis_/I_UV_ ratio exhibited a systematic increase with the amount of BiVO_4_ in the SILAR series, reaching a maximum for TiO_2_@S40. At this point, BiVO_4_ formed a nanostructured discontinuous coating on the TiO_2_ substrate. For the drop-casting TiO_2_@d electrode, the I_vis_/I_UV_ ratio is the highest because the compact BiVO_4_ layer strongly screens the contribution of TiO_2_ in the near UV; however, the absolute visible light photocurrent is the lowest. This finding underscores the significance of sufficient interfacial coupling between TiO_2_ and BiVO_4_, as depicted in S-scheme diagram, for efficient charge separation and extraction. From a chemical perspective, this interaction can be characterized as an interfacial contact between TiO_2_ and BiVO_4_, facilitated by an oxide bridge (Ti-O-Bi and Ti-O-V bridges). A schematic representation of this phenomenon is provided in the Supplementary Materials (see Figure S4) [65]. Conversely, the presence of excessively thick BiVO_4_ layers impedes transport and causes severe limitations in recombination. The BiVO_4_ synthesized in this work contains both monoclinic and tetragonal polymorphs. This phenomenon is corroborated by the optical data, which show that the powders exhibit two bandgap energies assigned to these phases (see Table 1). Moreover, the TiO_2_@BiVO_4_ electrodes manifest E_g_ values of 2.34 eV and 2.57 eV (see Figure 3d). Despite the fact that the BV-500 powder utilized for drop casting is abundant in monoclinic (see Table 1), the drop-casting electrode nevertheless provides the lowest photocurrent in visible light. This finding suggests that the morphology/effective thickness (transport and recombination losses in the dense surface layer) are the predominant factors in the PEC response under the prevailing conditions. The quantitative separation of the contribution of individual phases would require reference electrodes with pure phases and matched morphology, which is beyond the scope of this study.

Operational stability was also established based on current kinetics over time at a constant potential (0 V) for TiO_2_@S40 and TiO_2_@d photoanodes (see Figure S3 in Supplementary Materials). It has been demonstrated that both photoanodes manifest reproducible photocurrent responses to on-off illumination (370 nm) and maintain stability under measurement conditions throughout the duration of the test, despite an initial slight decrease in photocurrent intensity.

## 4. Materials and Methods

### 4.1. Reagents and Materials

Bismuth(III) nitrate(V) pentahydrate Bi(NO_3_)_3_∙5H_2_O (analytical reagent grade), ammonium metavanadate NH_4_VO_3_ (analytical reagent grade), nitric acid(V) 65% (chemically pure), sodium hydroxide (microgranules, analytical reagent grade), anhydrous ethyl alcohol 99.8% (analytical reagent grade), hydrochloric acid 35–38% (chemically pure) were purchased from Avantor Performance Materials Poland S.A. (Gliwice, Poland). Ti foil (0.127 mm, 99.7% trace metals basis) was purchased from Sigma-Aldrich (St. Louis, MO, USA).

### 4.2. Synthesis of BiVO_4_ Powders

The synthesis of bismuth vanadate powders was accomplished through the implementation of the precipitation method. Two precursor solutions were prepared. The first solution was a 0.02 M ammonium metavanadate, obtained by dissolving the appropriate mass of NH_4_VO_3_ in distilled water to a final volume of 0.5 dm^3^. The second solution was a 0.02 M bismuth(III) nitrate, prepared by thoroughly dissolving the salt in 2 M HNO_3_ to achieve a final volume of 0.5 dm^3^.

The vanadate solution was added dropwise to the Bi(NO_3_)_3_ solution, with continuous stirring, and then 4 M NaOH was added dropwise until pH = 3 was reached. The mixture was stirred throughout the reaction and for an additional 30 min. The precipitate was isolated from the solution through the repeated application of cycles of sedimentation, decantation, and washing with distilled water until a pH of 7 was attained.

Following a process of partial decantation, the suspension containing the precipitate was subjected to ultrasonication for a duration of five minutes in an ultrasonic bath, with the objective of desorbing HNO_3_. Subsequently, the suspension was subjected to a centrifugal force of 5000 rpm for a duration of 5 min. The rinsing and centrifugation sequence was repeated five times. The resultant solids were then subjected to a drying process at a temperature of 60 °C for a duration of 24 h. Following this, the dried solids were ground in an agate mortar. The resulting powder was then divided into four distinct parts: one was left unchanged (BV-ap), and the other three were heated in a tube furnace at 200 (BV-200), 350 (BV-350), and 500 °C (BV-500) for a period of 2 h, respectively. Subsequent to calcination, all powders were ground in an agate mortar.

### 4.3. Synthesis of TiO_2_@BiVO_4_ Heterostructures

To obtain TiO_2_@BiVO_4_ heterostructures, two methods were employed to deposit the BiVO_4_ layer on TiO_2_: drop-casting and successive ion layer adsorption and reaction (SILAR). The drop-casting method involves the application of BiVO_4_ powder suspension to the substrate in the form of drops, followed by a period of evaporation. In the SILAR method, the substrate undergoes alternation between immersion in cationic and anionic precursor solutions. The conditions for preparing the materials are summarized in Table 2. The schematic diagram of the process of obtaining TiO_2_@BiVO_4_ heterostructures is shown in Figure S1 in Supplementary Materials.

### 4.4. Synthesis of TiO_2_

Titanium foil substrates were prepared by cutting out 2 × 2 cm squares. The removal of the natural oxide layer was achieved through the etching of the titanium foils in concentrated HCl at 55 °C for a duration of 30 min. This was followed by a thorough rinsing with distilled water to ensure the complete removal of any residual chemicals. In order to facilitate the formation of a TiO_2_ surface layer under controlled conditions, the Ti foil was oxidized in a tube furnace at 600 °C for 7 h under forced atmospheric air flow (80 cm^3∙^min^−1^). Subsequent to the application of heat treatment, the samples were permitted to cool in a furnace to ambient temperature.

### 4.5. Drop-Casting Deposition

The BV-500 BiVO_4_ powder, having been previously synthesized and annealed, was deposited on TiO_2_ substrates by means of the drop-casting method. Prior to the deposition process, the BV-500 powder underwent a thorough grinding procedure, followed by ultrasonic treatment in ethanol. This treatment aimed to disintegrate micron-sized agglomerates and establish a stable suspension of nanocrystals. Subsequent to the solvent evaporation, a uniform layer of nearly spherical BiVO_4_ particles was formed on the TiO_2_ surface. The suspension was prepared in an ethanol solution by grinding 100 mg of BiVO_4_ with four drops of ethanol in an agate mortar. The resulting suspension was applied to the TiO_2_ surface and left to dry.

### 4.6. SILAR Deposition

To obtain further adsorption of the ionic layer and reaction deposition, two precursor solutions were prepared: solution I (0.01 M) by dissolving Bi(NO_3_)_3_ in 2 M HNO_3_ and solution II (0.01 M) by dissolving NH_4_VO_3_ in distilled water. The deposition process was executed in repeated cycles comprising four successive immersion steps: in (i) Bi(NO_3_)_3_ solution, (ii) distilled water, (iii) NH_4_VO_3_ solution, and (iv) is distilled water again. During the deposition process, both the solutions and the rinsing water were subjected to magnetic stirring. The duration of each immersion in each solution/water was 30 s per cycle. The total number of SILAR cycles applied ranged from 5 to 40. Subsequent to the deposition of BiVO_4_ via the SILAR method, no annealing was implemented. Following the SILAR cycles, the electrodes were thoroughly rinsed with deionized water and dried at room temperature prior to characterization and PEC measurements.

X-ray diffraction (XRD) was performed on a PANalytical Empyrean diffractometer (Malvern, UK) equipped with a copper anode (Cu Kα_1_, λ = 0.15406 nm). The diffraction data were analyzed using X’Pert HighScore Plus software (v.5.1) and the PDF-2 database. Raman spectroscopy was carried out on a Thermo Scientifi DXR3 (Waltham, MA, USA) confocal Raman microscope with a green laser (λ = 532 nm), in the range of 100–3500 cm^−1^ range. Surface morphology was examined using a Thermo Scientific Apreo 2S scanning electron microscope (SEM) equipped with an energy-dispersive X-ray spectroscopy (EDS) detector for elemental analysis. Morphology was also assessed with a Thermo Scientific DXR3 confocal Raman microscope in parallel with Raman spectral measurements. Spectral dependences of total reflectance were recorded using a Jasco V-670 UV-vis-NIR spectrophotometer (Hachioji, Japan) equipped with a 150 mm integrating sphere. Band-gap energies were determined using the reflectance differential calculation. The photoelectrochemical properties of the TiO_2_@BiVO_4_ heterostructured electrodes were evaluated in a three-electrode photoelectrochemical cell. The sample served as the working electrode, a platinum electrode coated with platinum black was used as the counter electrode, and a saturated calomel electrode (SCE) acted as the reference. The measurement setup comprised an electrochemical cell, a 450 W Xenon lamp as a light source with monochromator, a M161 potentiostat (MTM-ANKO, Kraków, Poland), and an EA Lab control software (v. 2.1, MTM-ANKO, Kraków, Poland). All measurements were made in aqueous electrolyte of 0.8 M Na_2_SO_4_.

## 5. Conclusions

The findings of this study demonstrate that the properties of BiVO_4_ can be effectively controlled through the application of heat treatment. The increase in crystallinity that occurs with increasing annealing temperature has a direct impact on the optical properties of the material and its suitability for use in photoelectrodes. This study determined that the method of depositing BiVO_4_ on TiO_2_ is of equal importance, as it determines the morphology and effective thickness of the surface layer. This, in turn, determines the type and magnitude of the photoelectrochemical reaction. The most favorable outcomes are attained with thin, nanostructured, discontinuous BiVO_4_ coatings, which facilitate charge separation and transport. Conversely, the presence of excessively thick layers imposes limitations on transport processes and results in an augmentation in recombination, consequently diminishing efficiency. Increasing the amount of BiVO_4_ shifts the photoanode reaction from the UV range towards the visible range. However, achieving this shift requires a balance between increased absorption and minimized losses. A general trend has been observed that is consistent with heterojunction-mediated charge separation, while the band diagram is treated as a working framework based on research values. The former effect is most commonly described as an S-scheme configuration, while the latter is occasionally termed a type II configuration.

## Figures and Tables

**Table 1:** Parameters of the crystal and electronic structure of as-prepared and annealed BiVO_4_ samples. * Amorphous-dominated with minor monoclinic and tetragonal fractions (below the XRD detection limit).

Sample	Phase	Phase Fraction XRD/Raman (%)	Crystal Size (nm)	E_g_ (eV)
BV-ap	amorphous *	100	-	2.36
				3.11
BV-200	amorphous *	100	-	2.35
				3.07
BV-350	monoclinic	49/57	19.3	2.30
	tetragonal	51/43	13.6	2.63
BV-500	monoclinic	87/80	46.1	2.31
	tetragonal	13/20	37.3	2.57

**Table 2:** The preparation conditions for the materials.

Type	Sample	BiVO_4_ Deposition Method	Number of SILAR Cycles	BiVO_4_ Annealing Temperature (°C)
powders	BV-ap			
	BV-200			200
	BV-350			350
	BV-500			500
substrate	TiO_2_			
heterostructures	TiO_2_@S5	SILAR	5	
	TiO_2_@S8	SILAR	8	
	TiO_2_@S10	SILAR	10	
	TiO_2_@S20	SILAR	20	
	TiO_2_@S30	SILAR	30	
	TiO_2_@S40	SILAR	40	
	TiO_2_@d	drop-casting		500

## Data Availability

The data supporting the findings of this study are available from the corresponding author upon reasonable request.

## Supplementary Materials

The following supporting information can be downloaded at: https://www.mdpi.com/article/10.3390/molecules31020353/s1, Table S1. Analysis of Raman modes positions. Table S2. Band-gap values determined in this work and representative literature ranges of band-edge positions for TiO_2_ and BiVO_4_ in aqueous media (potentials vs. NHE). Figure S1. Schematic diagram of the process of TiO_2_@BiVO_4_ heterostructures preparation and their morphology. Figure S2: Surface images of BiVO_4_ powders: as-prepared (a) and annealed at 200 °C (b), 350 °C (c), and 500 °C (d). Images were acquired with a confocal microscope at a magnification of 50×; Figure S3. Repeated current-time characteristic of TiO_2_@d and TiO_2_@S40 heterostructures under repeated light on/off cycles. Figure S4. Schematic representation of interfacial chemical structure.
