# Supplementary material for: Loading-Controlled Photoactivity in TiO2@BiVO4 Heterostructures
Source: Molecules. 2026 Jan 19;31(2):353. doi: 10.3390/molecules31020353 (PMC12844246; doi:10.3390/molecules31020353)
Supplement: Supplementary file 1 [file molecules-31-00353-s001.zip › molecules-4052127-supplementary.pdf]

Supplementary materials
on

Loading-Controlled Photoactivity in TiO_2@$BiVO_4$ Heterostructures

Małgorzata Knapik[1], Wojciech Zając[2], Agnieszka Wojteczko[1], Anita Trenczek-Zając[1*]

[1]Faculty of Materials Science and Ceramics, AGH University of Krakow, al. Mickiewicza 30, 30-059 Krakow, Poland
[2]Faculty of Energy and Fuels, AGH University of Krakow, al. Mickiewicza 30, 30-059 Krakow, Poland
* anita.trenczek-zajac@agh.edu.pl

Synthesis

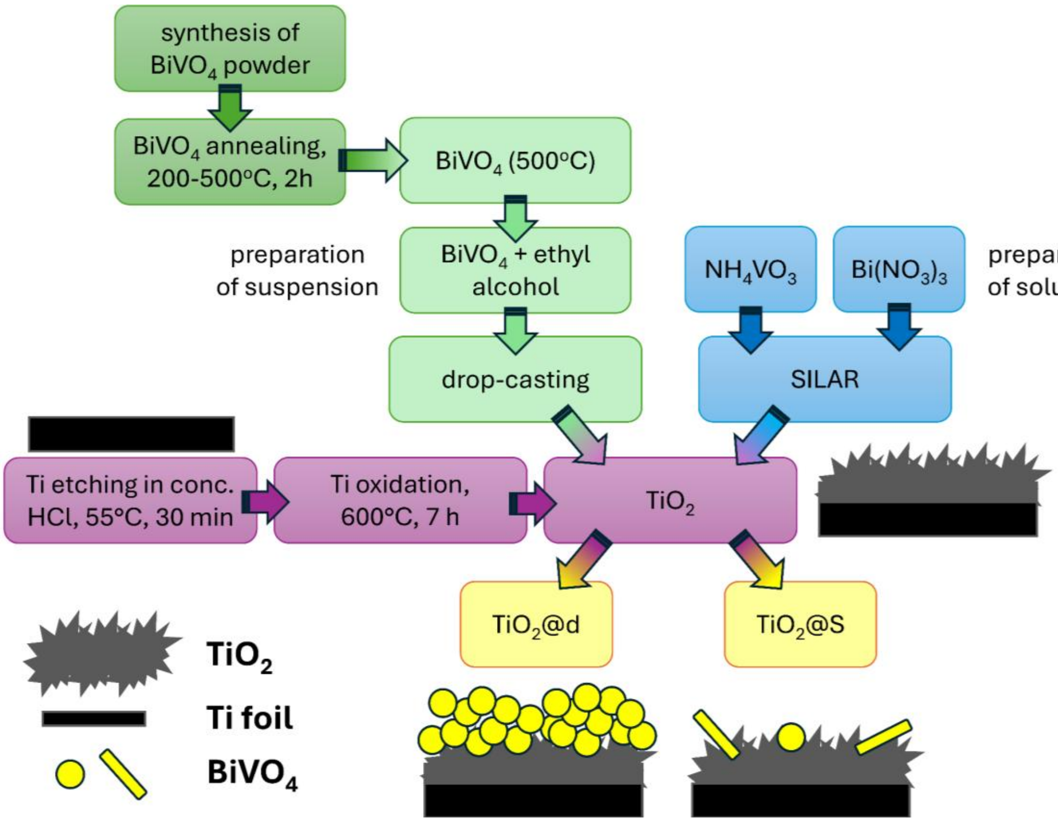

Figure S1. Schematic diagram of the process of TiO_2@$BiVO_4$ heterostructures preparation and their morphology.

Results and Discussion
Bismuth Vanadate Powders

Figure S2 compiles confocal microscopy images of bismuth vanadate powder surfaces after annealing at various temperatures. Each sample shows irregular particle shapes and a broad size distribution, with grains ranging from a few micrometers to

several tens of micrometers. In the BV-350 and BV-500 samples, the crystallite shapes are more clearly delineated, which may be related to a higher degree of crystallinity. A gradual change in image color is also apparent, which can be attributed to a transition from the amorphous state through a partially crystallized structure to a highly crystalline material with reduced light scattering.

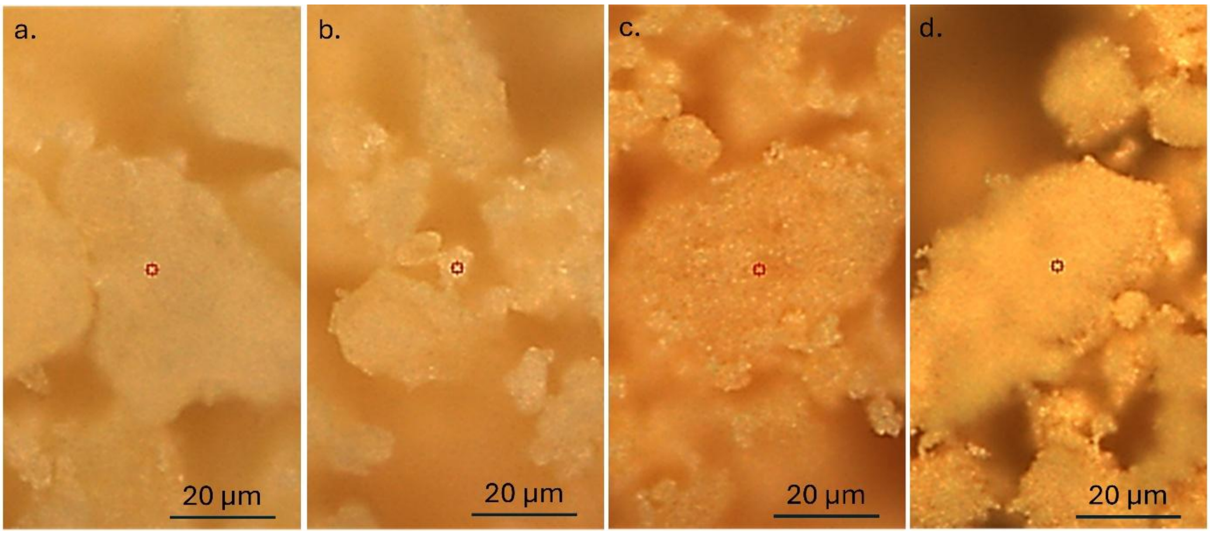

Figure S2. Surface images of $BiVO_4$ powders: as-prepared (a) and annealed at 200°C (b), 350 °C (c), and 500 °C (d). Images were acquired with a confocal microscope at a magnification of 50x.

TiO_2@$BiVO_4$ heterostructures

Table S1. Analysis of Raman modes positions.

raman shift (cm^{-1})	phase	assignment	reference
$BiVO_4$			
106.6-120.7	monoclinic/tetragonal	lattice (external) modes	[1,2]
137.2-150.3	monoclinic/tetragonal		
197.0-205.9	monoclinic/tetragonal		
324.8-338.0	monoclinic	VO_4 bending	[1,2]
358.5-370.0	monoclinic	VO_4 bending	[1,2]
692.0-710.0	monoclinic	V-O antisymmetric stretching	[1]
770.0-775.0	tetragonal	V-O antisymmetric stretching	[1]
811.0-815.0	monoclinic	V-O symmetric stretching	[1]
847.0-850.0	tetragonal	V-O symmetric stretching	[1]
TiO_2			
140	anatase/rutile	symmetric bending of O-Ti-O (anatase) / translational vibrations of a lattice involving Ti (rutile)	[3,4]
202	anatase	lattice or internal bending	[3,4]

239	rutile	combinations or overtones (2nd order)	[4,5]
324	rutile/lower titanium oxides eg. Ti_6O	combinations (2nd order in rutile) / -	[6]
444	rutile	planar O-Ti-O bending	[4]
512	anatase	symmetric stretching of Ti-O	[3,4]
606	rutile	symmetric stretching of Ti-O	[3,7]

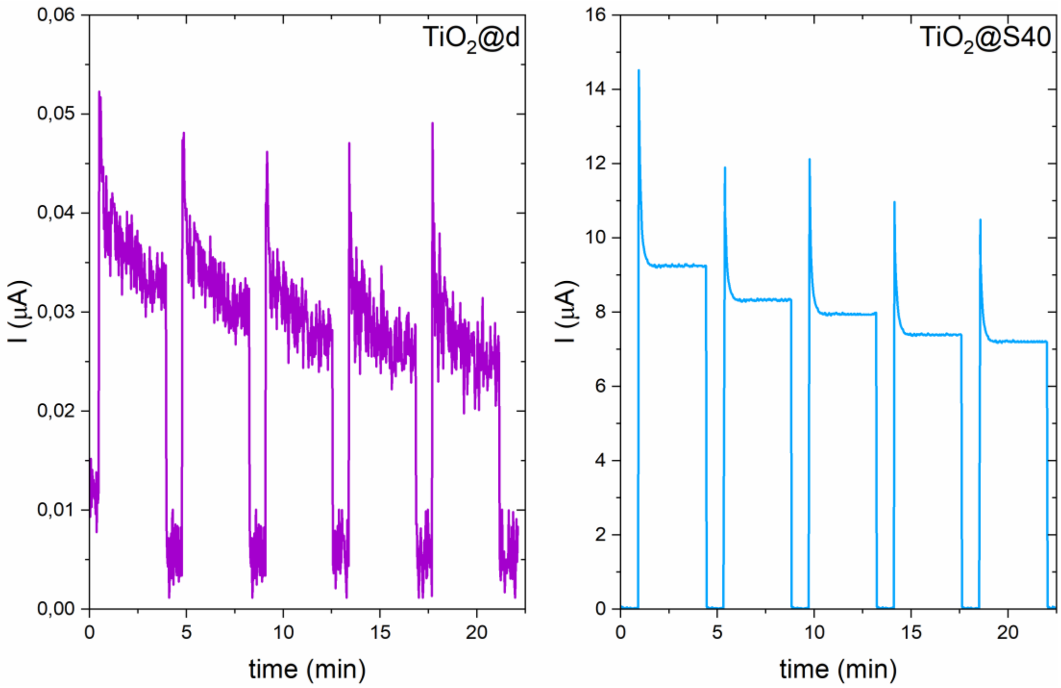

Figure S3. Repeated current-time characteristic of TiO_2@d and TiO_2@S40 heterostructures under repeated light on/off cycles.

Table S2. Band-gap values determined in this work and representative literature ranges of band-edge positions for TiO_2 and $BiVO_4$ in aqueous media (potentials vs NHE).

Material/phase	E_g (eV) this work	E_{CB}	E_{VB}	Note	Ref.
TiO_2	3.18	-0.2 to -0.4	2.8 to 3.0	typical values in neutral electrolytes	[8,9]
$BiVO_4$ (monoclinic)	2.34	0.0 to 0.4	2.4 to 2.8	reported variability with synthesis/defects	[8,10]
$BiVO_4$ (tetragonal)	2.57	-0.39	2.47	reported variability with synthesis/defects	[11]
H^+/H_2 redox level	-	0.00	-	reference level	
O_2/H_2O redox level			1.23	reference level	

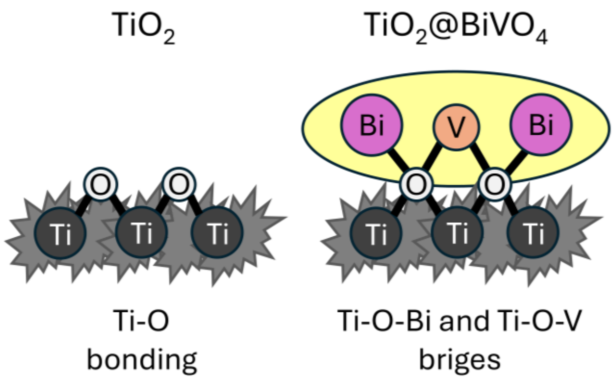

Figure S4. Schematic representation of interfacial chemical structure.

References

1. Dabodiya, T.S.; Selvarasu, P.; Murugan, A.V. Tetragonal to Monoclinic Crystalline Phases Change of BiVO 4 via Microwave-Hydrothermal Reaction: In Correlation with Visible-Light-Driven Photocatalytic Performance. *Inorg. Chem.* **2019**, *58*, 5096–5110, doi:10.1021/acs.inorgchem.9b00193.
2. Frost, R.L.; Henry, D.A.; Weier, M.L.; Martens, W. Raman spectroscopy of three polymorphs of BiVO 4 : clinobisvanite, dreyerite and pucherite, with comparisons to (VO 4) 3 -bearing minerals: namibite, pottsite and schumacherite. *J. Raman Spectrosc.* **2006**, *37*, 722–732, doi:10.1002/jrs.1499.
3. Ohsaka, T.; Izumi, F.; Fujiki, Y. Raman spectrum of anatase, TiO2. *J. Raman Spectrosc.* **1978**, *7*, 321–324, doi:10.1002/jrs.1250070606.
4. Balachandran, U.; Eror, N.G. Raman spectra of titanium dioxide. *J. Solid State Chem.* **1982**, *42*, 276–282, doi:10.1016/0022-4596(82)90006-8.
5. Sacco, A.; Mandrile, L.; Tay, L.-L.; Itoh, N.; Raj, A.; Moure, A.; Del Campo, A.; Fernandez, J.F.; Paton, K.R.; Wood, S.; et al. Quantification of titanium dioxide (TiO 2) anatase and rutile polymorphs in binary mixtures by Raman spectroscopy: an interlaboratory comparison. *Metrologia* **2023**, *60*, 055011, doi:10.1088/1681-7575/acf76d.
6. Zhang, M.-S.; Yin, Z.; Chen, Q.; Xijun, W.; Xiaoli, J. Raman scattering by nanophase titanium dioxide. *Ferroelectrics* **1995**, *168*, 131–137, doi:10.1080/00150199508007856.
7. Nicola, J.H.; Brunharoto, C.A.; Abramovich, M.; Conçalves da Silva, C.E.T. Second order Raman spectrum of rutile TiO 2. *J. Raman Spectrosc.* **1979**, *8*, 32–34, doi:10.1002/jrs.1250080108.
8. Resasco, J.; Zhang, H.; Kornienko, N.; Becknell, N.; Lee, H.; Guo, J.; Briseno, A.L.; Yang, P. TiO 2 /BiVO 4 Nanowire Heterostructure Photoanodes Based on Type II Band Alignment. *ACS Cent. Sci.* **2016**, *2*, 80–88, doi:10.1021/acscentsci.5b00402.
9. Zhou, P.; Wu, J.; Yu, W.; Zhao, G.; Fang, G.; Cao, S. Vectorial doping-promoting charge transfer in anatase TiO2 {001} surface. *Appl. Surf. Sci.* **2014**, *319*, 167–172, doi:10.1016/j.apsusc.2014.05.045.
10. Xiao, B.-C.; Lin, L.-Y.; Hong, J.-Y.; Lin, H.-S.; Song, Y.-T. Synthesis of a monoclinic BiVO 4 nanorod array as the photocatalyst for efficient photoelectrochemical water oxidation. *RSC Adv.* **2017**, *7*, 7547–7554, doi:10.1039/C6RA28262H.

11. Dai, D.; Liang, X.; Zhang, B.; Wang, Y.; Wu, Q.; Bao, X.; Wang, Z.; Zheng, Z.; Cheng, H.; Dai, Y.; et al. Strain Adjustment Realizes the Photocatalytic Overall Water Splitting on Tetragonal Zircon BiVO 4. *Adv. Sci.* **2022**, *9*, 1–10, doi:10.1002/advs.202105299.
